# RE: Rural-urban disparities and trends in cancer screening: an analysis of Behavioral Risk Factor Surveillance System data (2018–2022)

**DOI:** 10.1093/jncics/pkag063

**Published:** 2026-06-19

**Authors:** Neill Bates

**Affiliations:** Brody School of Medicine, Department of Academic Affairs, East Carolina University, Greenville, NC, United States

I read with great interest the article by Benavidez et al. examining contemporary trends in up-to-date breast, cervical, and colorectal cancer screening across the US adult population and potential cancer screening disparities between rural and urban communities.^1^ The analysis relied on data from the Centers for Disease Control and Prevention’s Behavioral Risk Fator Surveillance System (BRFSS), a nationally representative telephone survey designed to capture comprehensive information on clinical preventive health practices and risk behaviors of the US population.^2^ Consistent with current guidelines, Benavidez et al. defined cervical cancer screening as receipt of Papanicolaou (Pap) testing within the past 3 years at the time of the survey for women 21 to 65 years old or human papillomavirus (HPV) testing within the past 5 years for women 35 to 65 years old.^1^^,^^3^ The authors noted that the weighted prevalence of guideline adherent cervical cancer screening declined from 82% in 2018 to 79% in 2020, and it fell to 47% in 2022.^1^ Compared with women in urban areas, women in rural areas had decreased odds of being up to date with cervical cancer screening from 2018 to 2020, but this association was not significant in 2022.^1^ These findings may be a product of restrictions in access to primary care during the COVID-19 pandemic, which spurred temporary disruptions to preventive care services in both rural and urban populations.^1^^,^^4^ However, these findings, particularly the pronounced decline in cervical cancer screening in 2022, might be an artifact of survey respondents’ health literacy and changes in how cervical cancer screening was ascertained by BRFSS.

Previous cycles of BRFSS separately asked respondents whether they have had a Pap test or HPV test for cervical cancer screening, but, starting in 2022, BRFSS transitioned to asking respondents whether they have ever had a cervical cancer screening test and if they affirmed having cervical cancer screening, whether it was a Pap or HPV test. Low health literacy has been associated with decreased cervical cancer knowledge and subsequent screening; respondents may not realize Pap testing is a method of cervical cancer screening.^5^^,^^6^ This may suggest that current cycles of BRFSS may not reflect actual cervical cancer screening behavior. The results produced by Benavidez et al. must be interpreted with caution due to the methodological changes by BRFSS. Using data from 2018 to 2024 BRFSS with mirrored cervical cancer screening definitions to Benavidez et al.,^1^ the weighted prevalence of cervical cancer screening was significantly higher in the period preceding the change in survey questions compared with the period after (81% vs 56%, *P* < .001; Table 1). However, cervical cancer screening prevalence has rebounded in 2024, suggesting that declining cervical cancer screening may be a product of disruptions in preventive care services during the COVID-19 pandemic and changes to the phrasing of cervical cancer screening questions by BRFSS. Nonetheless, contemporary estimates of cervical cancer screening may not be feasible using BRFSS considering these methodological changes.

**Table 1.** Weighted prevalence of cervical cancer screening before and after the change in the survey questionnaire and over the study period (*n* = 416 080).^a^

Variable	Weighted prevalence (%)	95% confidence interval	*P*
Period			<.001
Before change (2018 to 2020)	80.5%	80.2% to 80.8%	
After change (2022 to 2024)	56.1%	55.7% to 56.6%	
Year			<.001
2018	81.5%	81.0% to 82.1%	
2020	79.4%	78.8% to 80.0%	
2022	46.6%	46.0% to 47.2%	
2024	65.5%	64.8% to 66.1%	

a Data analysis was performed using Stata/SE 19.5 (College Station, TX: StataCorp LP), and *P* < .05 was considered statistically significant.

## Data Availability

The data that support our findings are publicly available at the Centers for Disease Control and Prevention’s Behavioral Risk Factor Surveillance System at https://www.cdc.gov/brfss/index.html. Code that was written to analyze these data is available upon reasonable request.
